# Teleguided Point-of-Care Ultrasound for Fluid Assessment in Geriatric Inpatients Performed by Nurses and Medical Students: Prospective Observational Feasibility Study

**DOI:** 10.2196/88574

**Published:** 2026-06-12

**Authors:** Stephanie Heinemann, Mira Fleur Seitz, Christine A F von Arnim, Carolin Steinmetz, Miroslava Valentová

**Affiliations:** 1 Department of Geriatrics University Medical Center Göttingen Göttingen Germany; 2 DZHK (German Center for Cardiovascular Research), Partner Site Lower Saxony Göttingen Germany

**Keywords:** telemedicine, tele-ultrasound, point-of-care ultrasound, feasibility study, pilot study, geriatrics, remote supervision, digital health innovation

## Abstract

**Background:**

Fluid assessment in geriatric inpatients is challenging, as clinical signs are often unreliable. Inferior vena cava (IVC) ultrasound provides a rapid, noninvasive estimation of intravascular volume. Teleguided point-of-care ultrasound (POCUS) allows examiners without prior ultrasound experience to perform scans under real-time supervision.

**Objective:**

This study aimed to evaluate the feasibility, accuracy, efficiency, and user satisfaction of remote-guided IVC ultrasound performed by medical students and nurses without prior ultrasound experience in a geriatric inpatient setting.

**Methods:**

This prospective feasibility study was conducted between February and March 2025 in a geriatric inpatient ward at a German tertiary care hospital. Thirty hospitalized geriatric patients were recruited using a pragmatic convenience sampling approach on predefined study days. Each patient underwent 2 IVC ultrasound examinations (n=60) using a handheld device with TeleGuidance; one was performed by a medical student and one by a nurse. All scans were remotely supervised by an ultrasound-experienced cardiologist, who subsequently performed a third, independent IVC scan on each patient, serving as the reference standard. Examiners were 2 final-year medical students and 2 nurses, all without ultrasound experience, each performing 15 scans. Primary outcomes were technical feasibility (successful teleguidance connection), accuracy of IVC diameter measurement (≥80% within +2 mm to –2 mm), and examination duration (≤10 minutes). The secondary outcome was user satisfaction (≥75 on a 0-100 numeric rating scale).

**Results:**

Connectivity and remote supervision were consistently stable, enabling completion of all scans (feasibility 100%). IVC visualization was successful in 90% (27/30) of cases. Accuracy was achieved in 80% (48/60; 95% CI 67-88) of scans. Mean duration was 3.3 (SD 2.0) minutes. Mean user satisfaction was 89%, with all ratings ≥85%.

**Conclusions:**

Telemedicine-guided IVC ultrasound was feasible and well accepted in this geriatric inpatient setting. Nonexpert examiners were able to obtain clinically usable measurements under remote supervision within a few minutes after minimal training. These findings suggest that teleguided POCUS is a promising approach to support task sharing in geriatric care. Further studies are needed to confirm these results and to evaluate integration into clinical practice.

**Trial Registration:**

German Clinical Trials Register DRKS00035821; https://www.drks.de/search/de/trial/DRKS00035821/details

## Introduction

In geriatric inpatient care, timely and accurate assessment of fluid status is essential for guiding therapy. However, clinical signs are often misleading and may result in suboptimal treatment decisions. Recent evidence has shown that traditional bedside indicators (eg, skin turgor, tachycardia, and dry mucous membranes) have limited diagnostic value in older adults, whereas serum osmolality, history of low fluid intake, axillary dryness, and inferior vena cava (IVC) ultrasonography offer higher diagnostic accuracy [1,2]. Ultrasound assessment of the IVC provides a direct, rapid, and minimally invasive method for estimating intravascular volume and has been shown to be feasible for bedside use in older adults [3].

Beyond hospital settings, point-of-care ultrasound (POCUS) is increasingly used in decentralized care environments. In home medical care, POCUS improves access to diagnostic imaging but remains limited by training requirements and operator dependency [4]. Similarly, in hospital-at-home settings, POCUS has been shown to support clinical decision-making and avoid hospital transfer in a substantial proportion of cases [5]. These findings highlight both the potential of POCUS outside specialized settings and the challenges associated with its implementation.

With POCUS systems that integrate teleguidance functionality, experienced examiners can supervise in real time from a remote location while the person at the bedside positions the probe. This enables health professionals without formal ultrasound expertise—such as final-year medical students or nurses—to contribute effectively to diagnostic processes.

Previous studies have indicated that ultrasound examinations can be successfully performed under teleguidance even by individuals without prior ultrasound training. For example, patients themselves have been able to perform abdominal scans under remote supervision [6], and medical students have demonstrated comparable learning outcomes when guided by tele-ultrasound in training settings [7]. However, evidence on the feasibility and performance of teleguided POCUS under routine clinical conditions—particularly in geriatric inpatient populations—remains limited. In addition, teleguided POCUS depends on stable internet connectivity, which may limit consistent performance in certain clinical environments. Given the variability of in-hospital network conditions, connectivity was considered an integral component of real-world feasibility. To date, most studies have focused on training settings or selected populations rather than routine clinical care.

The aim of this pilot feasibility study was therefore to evaluate the implementation of teleguided POCUS IVC ultrasound under real-world clinical conditions, focusing on technical feasibility, measurement accuracy, efficiency, and user acceptance.

## Methods

### Design and Setting

We conducted an observational feasibility study at the Department of Geriatrics, University Medical Center Göttingen, between February and March 2025.

Reporting was informed by the STROBE (Strengthening the Reporting of Observational studies in Epidemiology) statement for observational studies. As this was an exploratory feasibility study, no formal sample size calculation was performed. The sample size was determined pragmatically based on feasibility considerations, including examiner availability and the number of patients that could be assessed within the study period. The study was deliberately designed to reflect routine clinical conditions, including pragmatic patient selection, variable in-hospital infrastructure, and minimal training of nonexpert examiners.

### Participants

Thirty hospitalized geriatric patients were recruited using a pragmatic convenience sampling approach on predefined study days to reflect routine patient flow and clinical feasibility rather than controlled selection. These study days were scheduled based on the simultaneous availability of the examiners (nurse, medical student, and supervising physician). On each study day, eligible patients currently admitted to the geriatric ward were approached for participation. Patients were eligible if they had provided written informed consent. Patients were excluded if the subcostal area was not accessible for ultrasound examination, for example, if it was covered by a dressing.

To avoid repeated measurements, patients who had already participated on a previous study day were not re-enrolled.

All eligible patients approached on study days agreed to participate, with the exception of 1 patient who declined due to personal circumstances. Patients who were temporarily unavailable during the study time frame (eg, due to diagnostic or therapeutic procedures, such as magnetic resonance imaging or dialysis) were not included.

### Examiners

Four examiners without prior ultrasound experience—2 final-year medical students and 2 registered nurses—each performed 15 examinations, resulting in 30 scans per group. Examiners were selected purposively based on availability, willingness to participate, and absence of prior ultrasound experience, to reflect the intended target group of nonexpert users. A board-certified physician with expertise in cardiology and sonography served as both the teleguidance supervisor and the reference examiner, performing an additional scan on each patient.

Before study initiation, all medical students and nurses received standard operating procedure–based training by the supervisor: 1 live demonstration of the IVC ultrasound on a patient (without the teleguidance function) and 2 supervised practice scans per person on patients (without the teleguidance function). The telemedicine feature was then introduced and practiced off-patient (ultrasound of a water-filled glove) prior to the first study scan. This session lasted approximately 10 to 15 minutes per participant and was intended to familiarize examiners with probe handling, image acquisition, and communication with the remote supervisor without involving patients. The standard operating procedure–based training took approximately 1 hour per person. The training was intentionally brief to reflect a realistic implementation scenario in routine care.

### System and Procedure

Ultrasound examinations were conducted using the Butterfly iQ+ handheld device (Butterfly Network, Inc) with integrated TeleGuidance functionality.

Examinations were conducted in the afternoon, approximately 1 hour after the lunch break, to ensure comparable measurement conditions across patients and to minimize interference with routine clinical care. Patients were examined in the supine position. The IVC was imaged from the subcostal view. Probe positioning was performed by the examiner under remote guidance; in some cases, patients were asked to briefly hold their breath to optimize image acquisition.

During each session, the supervising physician established a live audio-video connection, guided probe positioning, and measured the IVC maximum diameter directly on the remotely acquired images, using the average of 3 caliper measurements per case. Nonexpert examiners were responsible solely for probe positioning, while image interpretation and all measurements were performed by the supervising physician.

Each patient underwent 3 examinations: one performed by a medical student, one by a registered nurse, and one by the supervising expert, who served as the reference examiner. The same physician performed both the remote supervision and the reference examinations and was therefore not blinded to the teleguided scans.

To reduce potential bias, all teleguided examinations were completed first, followed by the reference examinations, which were conducted at the bedside and in a different order. The average time interval between teleguided and reference measurements was approximately 45 minutes per patient. We minimized changes in fluid status by performing examinations at rest and avoiding fluid boluses during the measurement window.

A study coordinator was present during all examinations and was responsible for time measurement, documentation, and pseudonymization of data. The coordinator ensured standardized examination conditions, including probe hygiene, appropriate patient positioning and coverage, and protection of patient confidentiality during remote supervision. A linkage file between patient identifiers and study codes was maintained by the coordinator.

After completion of all scans, participating nonexperts rated their overall user satisfaction on a 0 to 100 scale (0=poor, 100=very good) by answering the following question: “We would like to ask you to provide an overall evaluation of your experience using the Butterfly iQ+ ultrasound device and the TeleGuidance feature.”

### Outcomes

The primary end points were as follows:

- Technical feasibility of successful teleguidance connection (target 100%)
- Accuracy of IVC maximum diameter measurement compared with a reference scan (target ≥80% within ±2 mm)
- Duration of examination (target ≤10 minutes)

The secondary end point was as follows: user satisfaction (target ≥75 on a 0-100 numeric rating scale).

### Rationale for Predefined Thresholds

The predefined thresholds for feasibility (successful teleguidance connection), accuracy (≥80% of measurements within ±2 mm of the reference), examination duration (≤10 minutes), and user acceptance (≥75 on a 100-point scale) were selected as pragmatic minimum performance criteria for a clinically acceptable teleguided POCUS workflow in a geriatric inpatient setting.

As no established or standardized cutoffs exist for teleguided IVC ultrasound, these thresholds were defined a priori by the study team based on clinical experience and feasibility considerations. The ±2 mm accuracy margin corresponds to approximately 10% of the typical IVC diameter range and was considered an acceptable level of measurement deviation for clinical decision-making.

We deliberately did not assess IVC respiratory variation (collapsibility index) in this study, because it requires precise, time-critical image freezing at specific points of the respiratory cycle. This is more difficult under remote teleguidance due to occasional latency between bedside image acquisition and remote screen freezing, which could have introduced additional measurement error.

Accordingly, the thresholds function as feasibility benchmarks rather than evidence-based clinical decision limits.

### Analysis

Data were managed in REDCap (Research Electronic Data Capture). Statistical analyses were conducted using SPSS (version 28; IBM Corp). Descriptive statistics were calculated for all outcomes, including means and SDs for continuous variables and proportions for categorical variables. For proportion estimates (eg, accuracy rates), 95% CIs were calculated using the Wilson score method.

### Ethical Considerations

This feasibility study was approved by the local ethics committee of the University Medical Center Göttingen (application number 20/9/2024; December 10, 2024) and conducted in accordance with the Declaration of Helsinki. All participants (examiners and patients) provided written informed consent. Data from both examiners and patients were pseudonymized and stored in a secure REDCap database. No compensation was provided for participation in the study.

## Results

### Participants and Examiners

The study population comprised 30 geriatric inpatients (Table 1). Most patients were octogenarians with normal weight or overweight, moderate dependence in activities of daily living, and impaired gait. Mild cognitive impairment (Mini-Mental State Examination ≤24) was present in approximately 30% (9/30) of patients. Nearly half (14/30, 46.7%) of the patients had a history of heart failure.

The nonexpert examiners consisted of 2 registered nurses and 2 final-year medical students. The nurses were both female (mean age 40.5, SD 14.9 years) with a mean of 17.5 (SD 9.2) years of professional experience. The students included 1 male and 1 female (mean age 32.0, SD 5.7 years), both in their final year of medical school and had completed 4 months of clinical training on the geriatric ward during their practical year.

**Table 1:** Demographic and clinical characteristics of the study population (N=30).

	Values
Female sex, n (%)	16 (53.3)
Age (years), mean (SD)	82.8 (5.9)
BMI (kg/m^2^), mean (SD)	25.1 (4.8)^a^
MMSE^b^ (points), mean (SD)	26.0 (3.1)
TUG^c^ (seconds), mean (SD)	18.4 (7.5)^d^
Barthel index (points), mean (SD)	58.8 (19.9)
Heart failure, n (%)	14 (46.7)
Chronic obstructive pulmonary disease, n (%)	3 (10)

^a^n=26.

^b^MMSE: Mini-Mental State Examination [8].

^c^TUG: Timed Up and Go [9].

^d^n=21.

### Feasibility and Performance Outcomes

All examinations were conducted under routine ward conditions without modification of existing infrastructure or clinical workflow. All primary end points were met (Table 2). Technical feasibility was 100% (60/60). In 90% (27/30) of patients, the IVC could be visualized by all 3 examiners (supervisor, medical student, and nurse). In 3.3% (1/30) of cases, the IVC could not be visualized by any examiner. In the remaining 6.7% (2/30) of cases, the supervisor successfully visualized the IVC, while at least one of the nonexpert examiners could not (in one case, both; in one case, the nurse only). Overall visualization rate was 96.7% (29/30) for the supervisor, 93.3% (28/30) for the medical students, and 90% (27/30) for the nurses.

**Figure 1:**
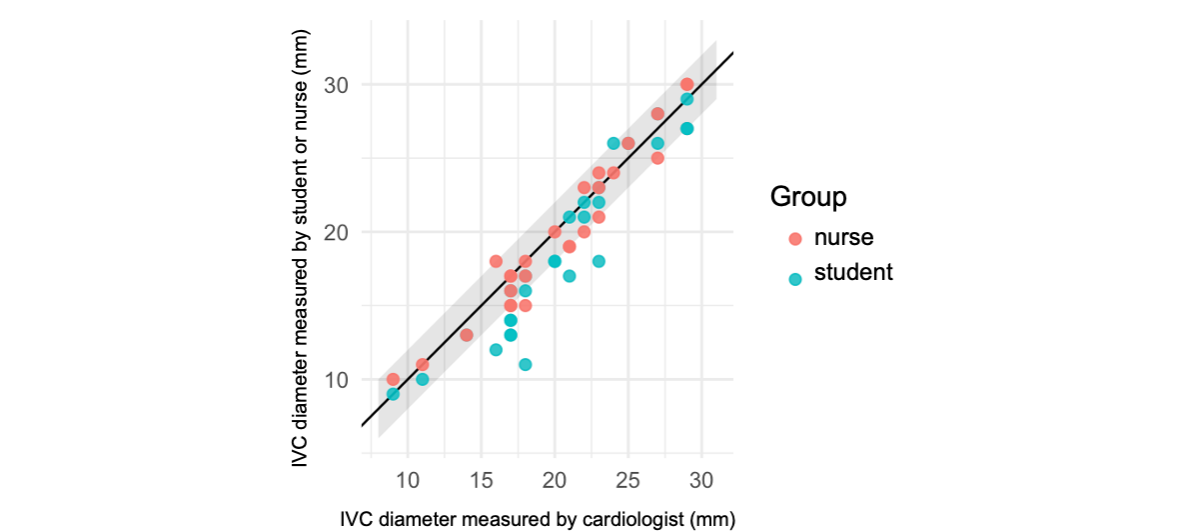
Agreement between remotely obtained inferior vena cava (IVC) diameter and the cardiologist reference measurement, showing the line of identity (black), the 2 mm tolerance interval (gray), and measurements by medical students (blue) and nurses (red), with 80% (48/60) of values falling within the tolerance band.

Of 60 scans, 80% (48/60; 95% CI 67-88) were within 2 mm of the reference standard examination (Figure 1). Greater variability was observed in smaller IVC diameters compared with larger values.

Mean examination duration for all nonexpert examiners was 3.3 (SD 2.0) minutes.

The secondary end point, user satisfaction, was also reached: mean satisfaction was 89 (range 85-94), exceeding the target threshold of 75.

**Table 2:** Overview of primary and secondary outcome measures.

Criterion		Values
**Primary outcomes**		
	Technical feasibility^a^ (n=60), n (%)	60 (100)
	IVC^b^ measurement accuracy^c^ (n=60), n (%)	48 (80)
	Examination duration (min), mean (SD)	3.3 (2.0)
**Secondary outcome**		
	User satisfaction^d^, mean (SD; range)	89 (85-94)

^a^Successful teleguidance connection.

^b^IVC: inferior vena cava.

^c^Measurements within 2 mm of the reference scan.

^d^Greater than or equal to 75 on a 0-100 numeric rating scale.

### Technical Challenges

The most relevant challenges during remote-guided examinations were related to communication and image acquisition. In particular, precise verbal guidance for probe positioning and fine adjustments proved demanding, especially during the first examinations performed by each examiner.

In addition, minor latency between image acquisition and remote screen freezing occasionally affected measurement accuracy. This was particularly noticeable in patients with low IVC diameters or respiratory variability, where optimal image capture required precise timing.

## Discussion

### Principal Findings

This study was designed to reflect real-world clinical conditions, including pragmatic patient recruitment, variable network performance, and minimal examiner training. Telemedicine-guided POCUS for assessing IVC measurements in geriatric inpatients was feasible, technically reliable, and well accepted, even when performed by users without prior ultrasound experience. Connectivity was assessed under routine clinical conditions, where network stability may vary within hospital environments. Remote-guided examinations met predefined targets for accuracy and examination duration, suggesting that nonexpert examiners were able to acquire images suitable for measurement under remote supervision.

Although no connection failures occurred (0/60, 0%), we observed minimal connection latency that hindered timely capture and exact measurement of the maximal IVC diameter in patients with marked respiratory variability. This may partly explain the lower agreement at smaller IVC diameters, as smaller IVCs tend to exhibit greater respiratory variation and are therefore more sensitive to delays when freezing the image remotely. Similar challenges related to transmission performance have been described in tele-ultrasound settings, where reduced frame rates impaired image interpretation and supervision [10]. In the future, this limitation may be mitigated by faster network technologies (eg, 5 G). Another potential solution would be to enable image freezing and frame review (scrolling) directly on the bedside device during remote sessions; this functionality was not available in the teleguidance mode used in our study.

Overall, measurement accuracy remained within predefined acceptable limits. These findings are particularly relevant given the known limitations of traditional clinical signs for fluid assessment in geriatric patients [1,2]. In our study, both medical students and nurses were able to perform IVC ultrasound under remote supervision after only minimal upfront training (approximately 1 hour), achieving a high level of measurement agreement. This supports the feasibility of task-sharing approaches and suggests that teleguided IVC-based fluid assessment can be extended beyond expert users.

These findings are consistent with prior tele-ultrasound studies demonstrating that remote supervision enables nonexperts to acquire and interpret ultrasound images with acceptable quality and confidence [11]. Although POCUS is increasingly recognized as a valuable diagnostic tool in geriatric care, its implementation remains limited, primarily due to a lack of training opportunities and availability of experienced examiners [1,12]. Teleguidance may help address these barriers by enabling real-time supervision of nonexpert examiners, even with minimal upfront training, thereby facilitating the use of ultrasound in settings where experienced examiners are not readily available.

### Limitations and Future Directions

This feasibility study has several limitations. The small sample size and monocentric design limit generalizability, and the controlled hospital environment may not fully reflect conditions in home or nursing home settings. The exploratory subgroup comparison between nurses and medical students was not powered for inferential analysis and should be interpreted with caution.

Another limitation is that the same expert (MV) served as both the teleguidance supervisor and the reference-standard examiner. Although separating these roles would have provided full independence of reference measurements, we intentionally used a single expert to avoid introducing additional between-examiner variability that could have obscured the performance of the inexperienced examiners. Nevertheless, this coupling of roles reduces the independence of the reference standard.

In addition, examiners received structured introductory training prior to study initiation, which may have facilitated probe handling under remote guidance; the feasibility of teleguided POCUS without prior training was not assessed. Patients included in the study were required to provide informed consent and tolerate the examination procedure, which may have led to underrepresentation of more severely impaired or uncooperative patients and may have favored patients with better sonographic accessibility, potentially inflating visualization success rates. The time interval between teleguided and reference examinations may have allowed for minor changes in volume status.

While these factors limit internal validity, they enhance external validity and reflect real-world implementation conditions in the geriatric clinical setting.

### Conclusions

In geriatric inpatients, telemedicine-guided IVC ultrasound was feasible and well accepted, and nonexpert examiners were able to obtain measurements under remote supervision. These findings support further evaluation of teleguided POCUS under real-world conditions to extend ultrasound-based fluid assessment beyond expert users. Given the exploratory design and limited sample size, further studies are needed to confirm these findings and to evaluate integration into clinical practice.

## Abbreviations

IVC: inferior vena cava
POCUS: point-of-care ultrasound
REDCap: Research Electronic Data Capture
STROBE: Strengthening the Reporting of Observational Studies in Epidemiology

## Data Availability

The datasets generated or analyzed during this study are available from the corresponding author on reasonable request.
